# Sensor-controlled fertigation management for higher yield and quality in greenhouse hydroponic strawberries

**DOI:** 10.3389/fpls.2024.1469434

**Published:** 2025-01-24

**Authors:** George Kerrigan Hutchinson, Lan Xuan Nguyen, Zilfina Rubio Ames, Krishna Nemali, Rhuanito Soranz Ferrarezi

**Affiliations:** ^1^ Controlled Environment Agriculture Crop Physiology and Production Laboratory, Department of Horticulture, University of Georgia, Athens, GA, United States; ^2^ Small Fruit Laboratory, Department of Horticulture, University of Georgia, Tifton, GA, United States; ^3^ Controlled Environment Agriculture Laboratory, Department of Horticulture & Landscape Architecture, Purdue University, West Lafayette, IN, United States

**Keywords:** *Fragaria × ananassa*, volumetric water content, energy use efficiency, water use efficiency, substrate, frequency domain reflectometry, capacitance sensor

## Abstract

Controlled environment agriculture (CEA) for strawberry (*Fragaria x ananassa*) production has experienced a growth in popularity in recent years, particularly in North America. One of the most common growing systems in CEA strawberry production is the soilless hydroponic system, which uses an inert substrate and nutrient solution to grow the plants. There are several strategies for water management in substrates, and most are based on a rigid schedule rather than variable plant water requirements over time. Comprehensive comparisons among the different strategies are lacking because they are often associated with complicated evapotranspiration models. The use of soil moisture sensors coupled with automated controllers that apply water when the substrate moisture drops below a set threshold has been proven efficient for select ornamental crops and citrus nursery crops but not for strawberries yet. This study aimed to compare various fertigation management strategies and, considering both yield and resource use, determine the optimal strategy for two newly released strawberry cultivars. ‘Florida Brilliance’ and ‘Florida Beauty’ were grown in a greenhouse hydroponic system under six different fertigation management strategies: one timer-based, one leaching fraction-based, and four sensor-based strategies that automatically applied fertilizer solution to maintain a constant volumetric water content threshold (0.36, 0.30, 0.225, or 0.15 m^3^·m^-3^). Yield and resource use were quantified during the 129-day experiment, and plants were harvested at the end of the experiment to measure biomass and foliar nutrients. The yield was used to calculate the water and energy use efficiencies for each strategy. Considering yield and resource use efficiencies, the two drier constant volumetric water content thresholds (0.225 and 0.15 m^3^·m^-3^) and the leaching fraction-based strategy had optimal performance. The results of this experiment can aid growers in employing more efficient fertigation management strategies to increase crop quality and reduce resource use for CEA strawberry production.

## 1 Introduction

Strawberries (*Fragaria x ananassa*) are a widely cultivated and popular fruit crop, with active commercial production in nearly a third of countries globally (Hytönen et al., 2018). While most domestic strawberries are grown using the annual hill plasticulture system, there is a growing interest in the United States and worldwide in using controlled environment agriculture (CEA) for strawberry production (Samtani et al., 2019). Greenhouse and vertical farming technologies (known as CEA facilities) can insulate crops from extreme weather, lengthen the growing season, and decrease the need for pesticides compared to field production, being a viable option to produce strawberries (Gómez et al., 2019).

CEA strawberries have been produced outside of the United States on a commercial scale for many years using tunnels and greenhouses in South Korea, Japan, the Netherlands, Belgium, France, the United Kingdom, and Italy (Ahn et al., 2021; Neri et al., 2012; Yoshida, 2013). The CEA strawberry industry is making its way into North America as well. A Dutch company recently completed a 29-hectare greenhouse facility for commercial strawberry production in Ontario, Canada, the largest such facility on the continent (Hemmes, 2021). Furthermore, in 2022, a large CEA company announced a partnership with a global leader in the strawberry market to build what will purportedly be the largest CEA complex in the world in Virginia that will produce millions of pounds of strawberries annually using hydroponic growing systems (Green, 2023; Oller, 2022).

The most common type of hydroponic system used to produce strawberries using CEA is a substrate culture system, also known as an aggregate or soilless culture system (Gómez et al., 2019). Substrate culture systems provide a soilless material, usually peat, coco coir, perlite, or some mix thereof, into which the crop’s roots can grow. The substrate can be irrigated using a nutrient solution containing fertilizers, which is called fertigation (a portmanteau of irrigation and fertilization), using drip emitters. There are several strategies to determine when and how much fertigation should be applied to the crops, most of them based on weather and soil parameters. The simplest and least expensive approach is to use a timer to regularly fertigate at a set amount at a set interval (Raviv et al., 2019). The most common and recommended strategy is regularly applying the fertigation solution until 20-30% of the applied volume leaches through the substrate (Kubota, 2019). An emerging approach is to use soil moisture sensors that can automatically activate irrigation based on real-time water uptake by the plants.

There are two common types of soil moisture sensors: tensiometers and dielectric capacitance sensors. Tube-shaped tensiometers contain a ceramic tip on one end and a pressure gauge on the other. They calculate matric potential by determining the tension experienced by moisture inside the tube, which is proportional to the wetness of the substrate (Raviv et al., 2019). The more popular type of soil moisture sensor for CEA is the dielectric capacitance sensor, also known as a capacitance sensor or a frequency domain reflectometry (FDR) sensor (Rhie and Kim, 2017). FDR sensors measure the dielectric permittivity of substrates by using parallel metal rods to analyze changes to the frequency domain of an electromagnetic pulse as it passes through the substrate (Raviv et al., 2019). FDR sensor-based fertigation has been successfully employed previously to increase crop quality while reducing water use and leachate waste in several ornamental and food crops in CEA production (Burnett and van Iersel, 2008; Ferrarezi et al., 2015; Nemali and van Iersel, 2006; Palumbo et al., 2021; van Iersel et al., 2009). Previous studies in CEA strawberry production have explored soil moisture sensor-based fertigation. These studies have mainly focused on comparing different soil moisture thresholds or comparing sensor-based fertigation management strategies to the simple but outdated timer-based fertigation (Bonelli et al., 2024; Choi et al., 2016, 2021; Cormier et al., 2020; Hoppula and Salo, 2007). However, few studies have compared sensor-based fertigation to timer-based fertigation and the commonly employed leachate fraction fertigation, especially in strawberries.

This experiment aimed to compare several soil moisture-based fertigation strategies to a timer-based and a leachate fraction fertigation in hydroponically grown strawberries and measure the differences in strawberry fruit yield and quality. Additionally, we quantified the resource inputs (i.e., water and energy) over the growth cycle and calculated the resource use efficiencies concerning the output, i.e., yield, to determine the optimal fertigation management strategy for CEA strawberry production.

## 2 Materials and methods

### 2.1 Location and environmental conditions

This experiment was conducted at the University of Georgia (College of Agricultural and Environmental Sciences, Department of Horticulture, Controlled Environment Agriculture Crop Physiology and Production laboratory) in Athens, Georgia, United States (latitude 33°55’55.10” N, longitude 83°21’50.51” W, altitude 198 m) from December 2022 to April 2023 in a polycarbonate greenhouse with controlled conditions.

Greenhouse air temperature and relative humidity were monitored using a digital sensor (HMP60; Vaisala, Helsinki, Finland) connected to a datalogger (CR1000X; Campbell Scientific, Logan, UT, United States) for automatic data collection. Average ± standard error day and night temperatures were 23.3 ± 0.04 and 18.2 ± 0.02°C, respectively. Day and night relative humidities were 40.5 ± 0.25 and 53.4 ± 0.24%, respectively. Vapor pressure deficit (VPD) was calculated using this temperature and relative humidity data and were 1.8 ± 0.01 and 0.96 ± 0.004 kPa for day and night, respectively. Ambient sunlight was augmented with light-emitting diode (LED) fixtures (SPYDRx; Fluence Bioengineering, Austin, TX, United States), which were controlled by a digital timer (Model 26898; Jasco Products LLC, Oklahoma City, OK, United States) to be activated from 4:00 PM to 7:30 PM daily. Canopy-level light was measured by a quantum sensor (SQ-610; Logan, UT, United States) connected to a separate datalogger (CR1000; Campbell Scientific, Logan, UT, United States) and resulted in a mean daily light integral (DLI) of 17.5 ± 0.67 mol·m^-2^·day^-1^.

### 2.2 Plant material

Live plugs of strawberry cultivars ‘Florida Brilliance’ and ‘Florida Beauty’ propagated in a commercial nursery (Production Lareault, Lavaltrie, QC, Canada) arrived at the greenhouse in October 2022. Plants were watered and fertilized regularly, sorted, and transplanted in November 2022. Plants were also watered and fertilized uniformly until treatments began in December 2022.

### 2.3 Hydroponic system

The recirculating hydroponic system consisted of 24 18-L troughs measuring 99.5 × 19.5 × 12.5 cm (L × W × H) (Article #7418; Beekenkamp Verpakkingen, Maasdijk, Netherlands), resting on 218 × 17.5 × 7 cm (L × W × H) metal drainage gutters (B200 profile; Haygrove Limited, Ledbury, United Kingdom) with two troughs per gutter. The gutters drained into 121 L plastic reservoirs (H-3687; Uline, Pleasant Prairie, WI, United States) for recirculation. Four plants of each cultivar were placed in every trough in two contiguous lines of four (eight total plants). The substrate was a 1:1 (volume:volume) mixture of super coarse perlite (Horticultural Perlite; Whittemore Co., Lawrence, MA, United States) and a bark-based media mix (Metro-Mix 830; Sun Gro Horticulture, Agawam, MA, United States). The solution was delivered per trough via drip irrigation with four emitters (Catalog no. 22000; Netafim, Tel Aviv, Israel).

### 2.4 Fertilization

A modified Yamazaki fertilizer solution was used for fertigation in all systems (Kroggel and Kubota, 2017). The solution contained (all values in mg·L^-1^): 77 total nitrogen (N) with 74 nitrate-nitrogen (NO_3_-N) and 3 ammoniacal-nitrogen (NH_4_-N), 15 phosphorous (P), 120 potassium (K), 52 calcium (Ca), 12 magnesium (Mg), 17 sulfur (S), 0.34 boron (B), 0.5 copper (Cu), 2 iron (Fe), 0.55 manganese (Mg), 0.05 molybdenum (Mo), and 0.33 zinc (Zn).

### 2.5 Treatments

We tested six different fertigation management strategies: one timer-based, one leaching fraction-based, and four sensor-based strategies that automatically applied nutrient solution to maintain a constant volumetric water content (θ) threshold (0.36, 0.30, 0.225, or 0.15 m^3^·m^-3^), with four replications. Each treatment was randomly assigned to one group of four troughs, for a total of 24 troughs, and each group of four troughs was fertigated by a separate reservoir (H-3687; Uline, Pleasant Prairie, WI, United States) and pump (PE-1; Little Giant, Oklahoma City, OK, United States).

The timer-based treatment (“Timer”) used the datalogger and relay to activate the pump for three minutes every three hours throughout the experiment. The leaching-fraction-based treatment (“Leach”) was manually controlled and had a target of 20-30% leachate per fertigation event. The four thresholds for the θ treatments were selected based on fractions of the substrate container capacity (θ 0.42 m^3^·m^-3^) to represent a broad range of substrate moisture contents. The four θ thresholds were (from high to low) 0.36, 0.30, 0.225, and 0.15 m^3^·m^-3^, chosen based on previous experiments performed in our lab. These four treatments were monitored by a soil moisture sensor (GS3; METER Group, Pullman, WA, United States) positioned in the center of each trough. The soil moisture sensors (n = 24) were connected to a datalogger (CR1000X; Campbell Scientific, Logan, UT, United States) for automatic data collection and irrigation control. A relay (SDM-CD16AC; Campbell Scientific, Logan, UT, United States) connected to the datalogger was used for pump activation in all treatments.

### 2.6 Reservoir pH and electrical conductivity (EC) measurements

Leachate volume, pH, and EC were recorded after each fertigation event. The solution pH and EC were regularly measured with a digital probe (#HI98131; Hanna Instruments, Smithfield, RI, United States) and adjusted to maintain a range between 5.5 and 6.5 pH and 0.75 and 1.25 dS·m^-1^, respectively. A commercial product derived from phosphoric acid was used to reduce the solution pH (pH-Down; Advanced Nutrients, West Hollywood, CA, United States), while an 8M solution of potassium hydroxide was used to raise the solution pH. EC was lowered by diluting the solution with tap water.

### 2.7 Substrate θ and number of irrigations

The datalogger also automatically recorded θ measurements for all troughs every 15 minutes. The average of the four soil moisture sensor measurements in each treatment group was used to determine the number of irrigations or pump activations. If the average of the four θ measurements were below the treatment threshold, the pump would activate to fertigate all four troughs in the group for three minutes. The datalogger recorded the automatic pump activation time every 3 minutes throughout the experiment.

### 2.8 Fruit harvest measurements

Fruit harvests were conducted every other week between December 2022 and January 2023, then changed to every week for February through April 2023, with 15 harvests in total. This change was instituted to accommodate the larger fruit production as the season progressed and to minimize fruit losses due to fungal pathogens. Fruit that were 70% or more ripe were harvested from each measurement plant. Fruit from each plant were counted and collectively weighed using a digital scale (Item #30430061; Ohaus Corporation, Parsippany, NJ, United States) to measure the fresh fruit yield. Marketable fruit were also counted and collectively weighed to obtain the marketable yield. A fruit was considered marketable if it weighed at least 8 g and was nicely shaped (a sign was evenly pollinated). The largest marketable fruit (or simply the largest fruit if none were marketable) was cut longitudinally in half. One of the halves was weighed and crushed using cheesecloth and a garlic press to measure total soluble solids (TSS) using a digital refractometer (#HI96801; Hanna Instruments, Smithfield, RI, United States). The other half and the remaining fruit from the plant were placed in an 80°C oven for several days until completely dehydrated. The dehydrated fruit were weighed again to obtain the dry fruit biomass. By weighing the half-fruit used for TSS analysis, the total fruit biomass before and after dehydration was known, and thus, fruit water content could be calculated.

### 2.9 Plant harvest measurements

The strawberry plants were terminated on April 27, 2023 (129 days after transplanting). Before harvesting, the plant height was measured using a meter stick. Plants were harvested by cutting the crown at the soil line. Plants were weighed using a digital scale (Model #PB3002; Mettler Toledo, Griefensee, Switzerland) to determine fresh shoot biomass. The number of flowers, fruit, runners, and leaves was counted for each plant. Plant mortality was also assessed at this stage by direct counting.

The harvest index was calculated using the total fruit yield and the fresh shoot biomass: total fruit fresh weight ÷ (total fruit fresh weight + plant fresh weight). Next, all healthy trifoliate leaves for each plant were scanned using a leaf area meter (LI-3100; LI-COR, Lincoln, NE, United States) to obtain the total leaf area (LA). Each plant was placed into a paper bag and dried at an 80°C drying oven for several days. Dry shoot biomass was measured using the same digital scale.

Dried trifoliate leaves were placed in sample bags and sent to a commercial lab (Waters Agricultural Laboratories, Camilla, GA, United States) for tissue nutrient concentration analysis. Leaf N was determined by a high-temperature combustion process (Nelson and Sommers, 1973). Leaf P, K, Mg, Ca, S, B, Fe, Cu, Mn, and Zn concentrations were determined by inductively coupled plasma atomic emission spectrophotometer after wet acid digestion using nitric acid and hydrogen peroxide (Twyman, 2005).

### 2.10 System measurements and resource use quantification

All reservoir volumes were tracked throughout the experiment. Reservoirs were filled to a known volume at the start of the experiment and filled again to that known volume after draining and refilling. Residual reservoir volume was measured during drain and refill events, triggered when reservoir volume was low and/or when the reservoir pH and EC were out of the ideal range. By knowing reservoir volume before and after refills, total system losses due to evapotranspiration (ET) were calculated by simple subtraction.

To calculate plant water use efficiency (WUE), ET per plant was calculated by dividing reservoir ET by the number of plants supplied by that reservoir. Yield per plant was then divided by this ET per plant (based on from which reservoir the plant was fertigated) to obtain plant WUE in grams per liter.

Total system energy use was calculated by tracking the total pump activation time in hours for each fertigation management strategy. As previously mentioned, the datalogger recorded the automatic fertigation activations for each of the four θ threshold treatments throughout the experiment. The Timer treatment was activated at a regular interval of 3 minutes every 3 hours, and the Leach treatment fertigation events were manually timed. All treatments used the same pump model, which has a power consumption of 36 W per manufacturer specifications. The total pump run times and power consumption rate were multiplied to obtain total energy use in kilowatt hours (kWh) for each fertigation management strategy.

To calculate plant energy use efficiency (EUE), energy use per plant was first calculated by dividing the total treatment energy use by the total number of plants (32). Yield per plant was then divided by this energy use per plant to obtain plant EUE in grams per kWh.

### 2.11 Experimental design and statistical analysis

The study was arranged on a randomized block design, with six treatments and four replications. Statistical analysis was performed by one-way analysis of variance (ANOVA) with Tukey’s *post-hoc* test using statistical software (SigmaPlot Version 15; Systat Software, San Jose, CA, United States) to determine significant differences among treatments. When a data set did not meet the ANOVA’s normality or equal variance conditions, a Kruskal-Wallis test with Dunn’s *post-hoc* was conducted using the same statistical software. A probability (*P*) level of 0.05 was used in all tests. Results from each cultivar were analyzed separately.

## 3 Results

### 3.1 Reservoir pH and EC

**Figure 1:**
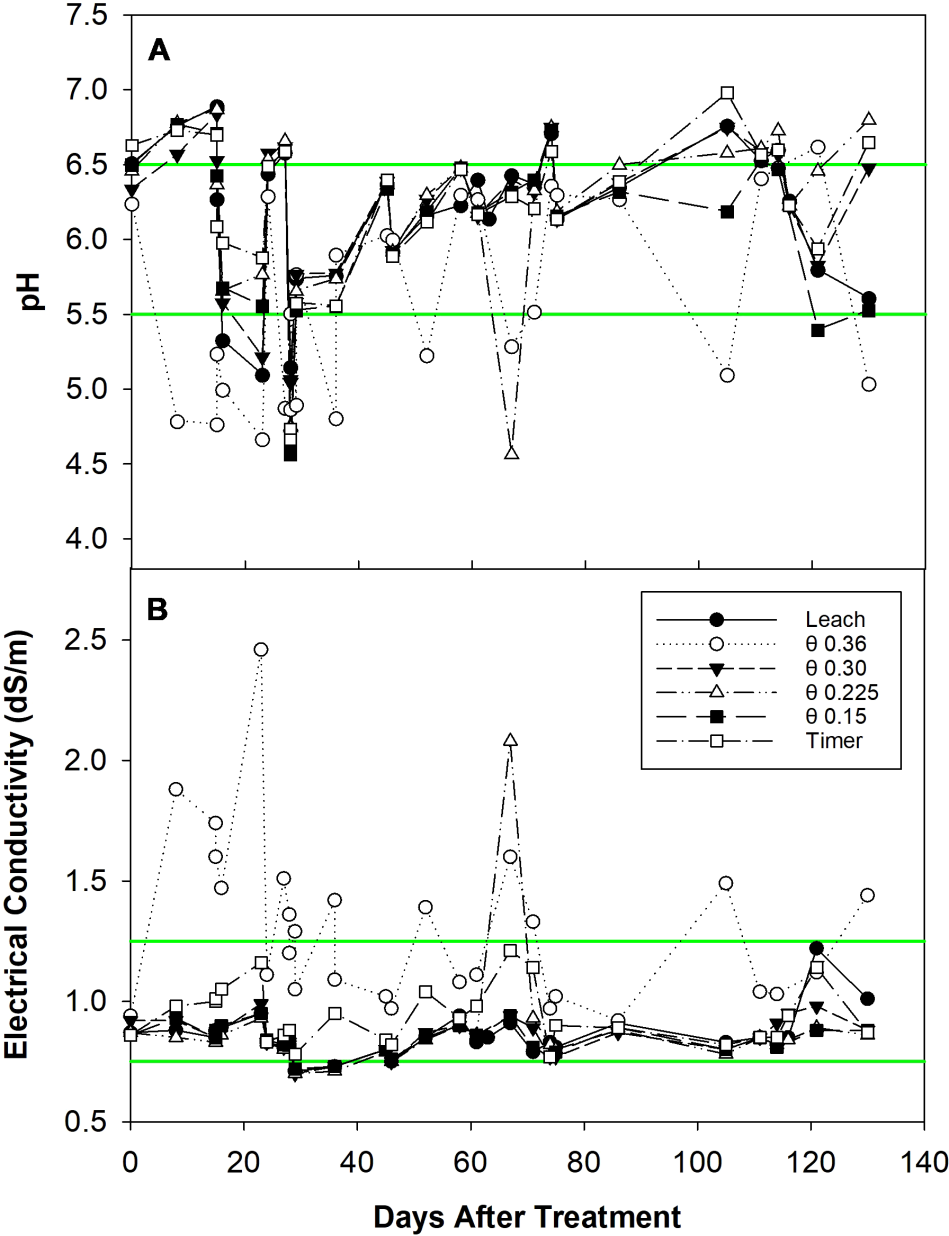
Reservoir pH **(A)** and electrical conductivity (EC) **(B)** for different fertigation management strategies from zero to 130 days after treatment. Individual data points represent pH or EC measurements. Green lines represent the bounds of the ideal range (5.5-6.5 for pH and 0.75-1.25 dS·m^-1^ for EC); data points between the green lines are considered within the range.

Reservoir pH was controlled successfully in all treatments during the experiment (**Figure 1A**). Notably, the θ 0.36 m^3^·m^-3^ treatment induced the greatest pH variation and deviated from the other five treatments. There is one extreme deviation in the θ 0.225 m^3^·m^-3^ treatment at 65 DATS. The average ± standard error measured reservoir pH was 6.2 ± 0.09, 5.6 ± 0.12, 6.2 ± 0.09, 6.2 ± 0.12, 6.1 ± 0.11, and 6.2 ± 0.10 for the Leach, θ 0.36, 0.30, 0.225, 0.15 m^3^·m^-3^, and Timer treatments, respectively. Reservoir EC was more uniform except in the θ 0.36 m^3^·m^-3^ treatment (**Figure 1B**). The extreme deviation at 65 DATS for the θ 0.225 m^3^·m^-3^ treatment previously mentioned can also be seen in this graph. Average ± standard error measured reservoir EC was 0.86 ± 0.017, 1.30 ± 0.063, 0.85 ± 0.014, 0.88 ± 0.046, 0.84 ± 0.011, and 0.94 ± 0.023 dS·m^-1^, respectively.

### 3.2 Pump activation and θ control

**Figure 2:**
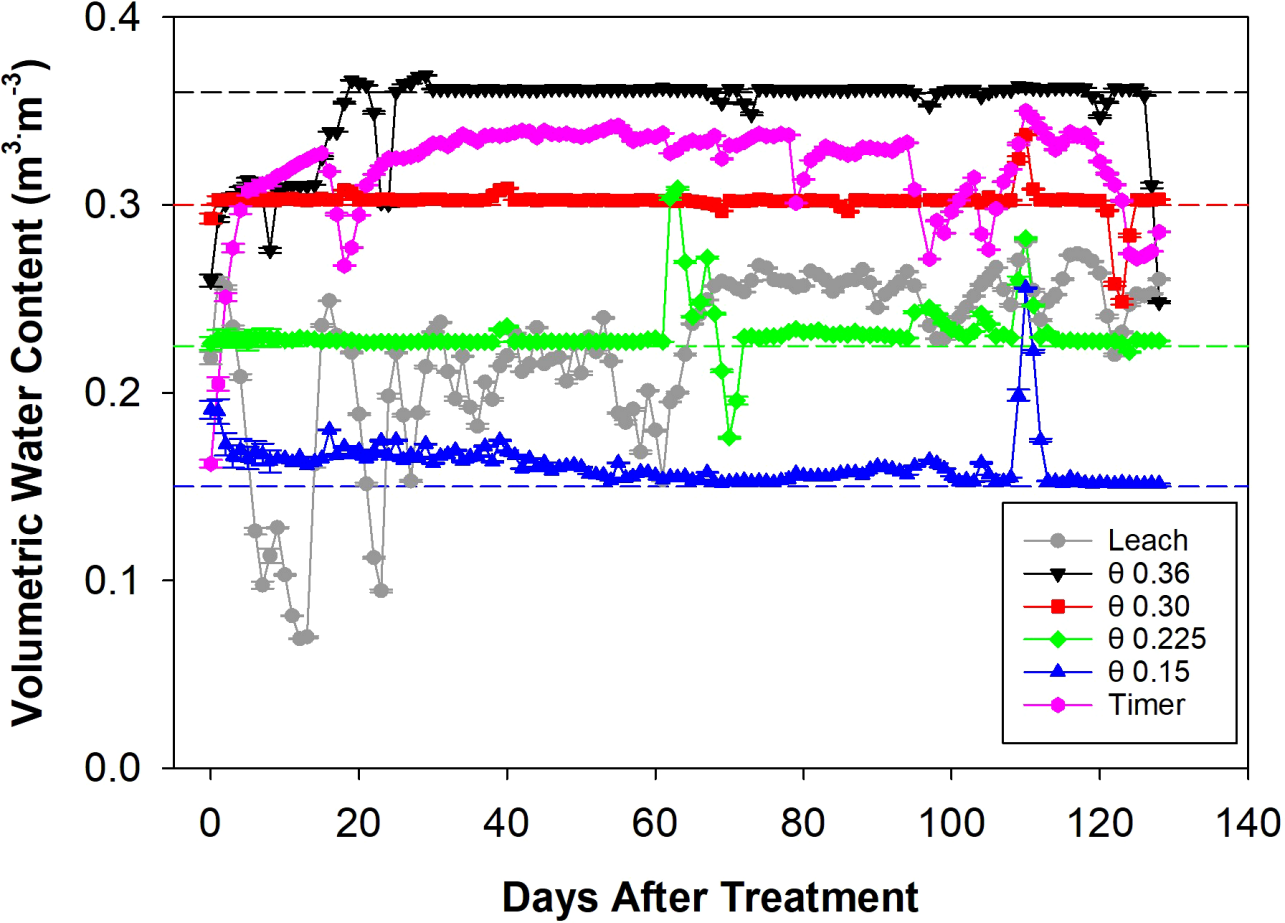
Daily volumetric water content (θ) data for different fertigation management strategies measured by the soil moisture sensors from zero to 130 days after treatment. Data points represent average ± standard error θ values for four troughs in 24 hours. Dashed lines represent θ target thresholds.

The treatments began at 10:00 AM on December 19, 2022, and ended at 10:00 AM on April 27, 2023. During this period, the θ 0.36 m^3^·m^-3^ treatment pump was active for 35.4% of the time, which was by far the highest activation rate of all the treatments. This was followed by the θ 0.30 and 0.225 m^3^·m^-3^ treatment pumps at 5.21% and 3.52% activation, respectively. The Timer treatment pump was active for 1.62% of the time, the Leach treatment pump was active for 1.44%, and the θ 0.15 m^3^·m^-3^ treatment pump had the lowest activation rate at 1.25%. These pump activation rates correspond to a total applied solution volume of 1,065 L for the Leach treatment, 26,212, 3,859, 2,605, and 972 L for the θ 0.36, 0.30, 0.225, and 0.15 m^3^·m^-3^ treatments, and 1,203 L for the Timer treatment. On average, each plant received a daily solution volume of 0.26 L in the Leach treatment, 6.35, 0.93, 0.63, and 0.22 L in the θ 0.36, 0.30, 0.225, and 0.15 m^3^·m^-3^ treatments, and 0.29 L in the Timer treatment. The sensor-activated pump control system consistently maintained the target θ thresholds throughout the experiment (**Figure 2**). Minor deviations can be seen for the θ treatments due to control component failures that were quickly repaired. These occurred from 0-25 DATS for the θ 0.36 m^3^·m^-3^ treatment, between 60 and 70 DATS for the θ 0.225 m^3^·m^-3^ treatment, and at 110 DATS for the θ 0.30 and 0.15 m^3^·m^-3^ treatments. The Leach treatment showed high variability during the first 70 days of the experiment, with this reducing during the second half. The Timer treatment resulted in a consistent and high θ level throughout the experiment.

### 3.3 Total and marketable yield

**Figure 3:**
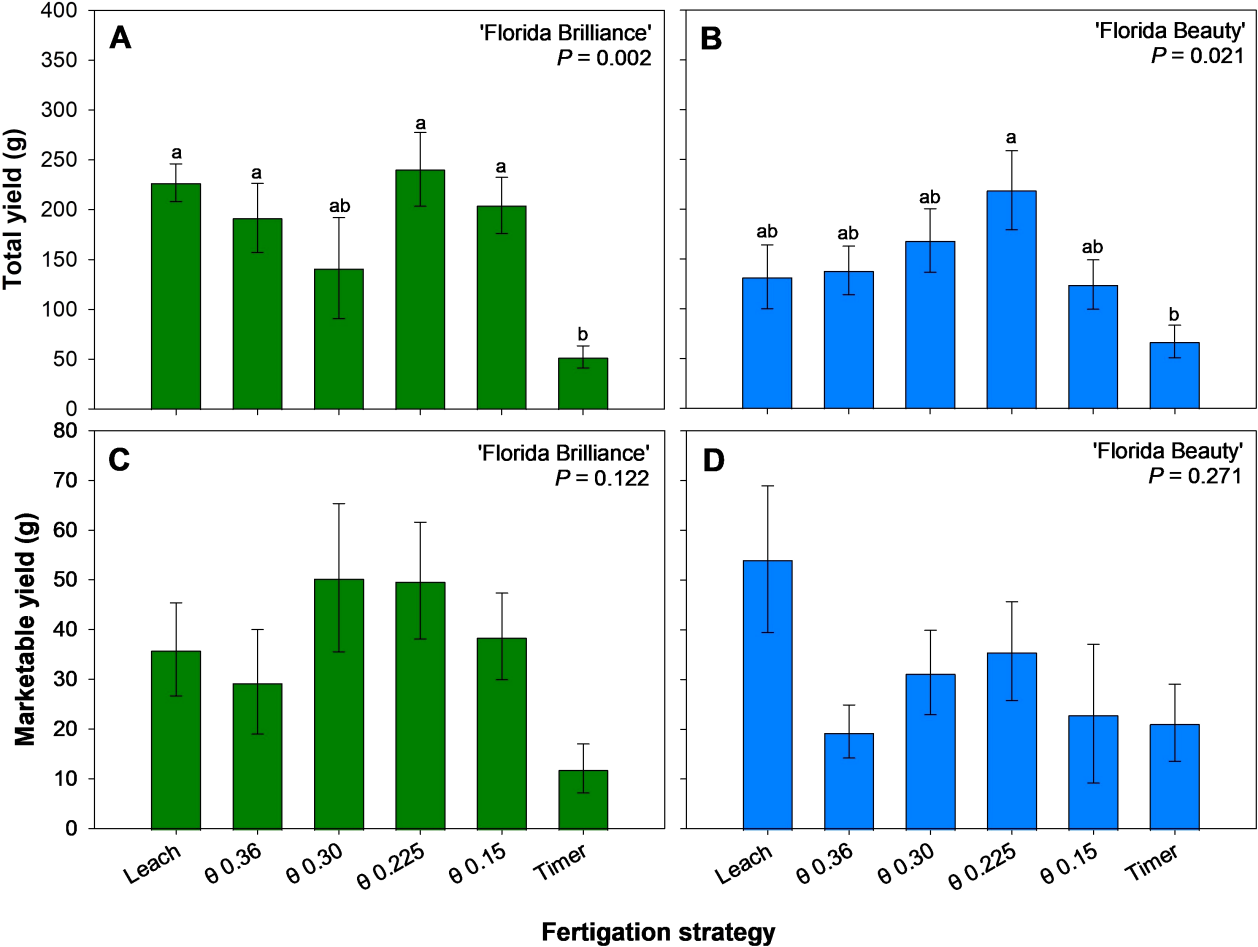
Total yield per plant for ‘Florida Brilliance’ **(A)** and ‘Florida Beauty’ **(B)**, and marketable yield per plant for ‘Florida Brilliance’ **(C)** and ‘Florida Beauty’ **(D)**. Each bar represents the average ± standard error of four replications with two measurement plants. Bars with the same letter show no significant difference; bars with different letters are statistically different at a 5% significance level (*P* < 0.05).

The Leach, θ 0.36, 0.225, and 0.15 m^3^·m^-3^ treatments resulted in at least 291% higher total yield than the Timer treatment for the ‘Florida Brilliance’ cultivar (*P* = 0.002) (**Figure 3A**). For ‘Florida Beauty’, the θ 0.225 m^3^·m^-3^ treatment resulted in a 227% higher total yield than the Timer treatment (*P* = 0.021) (**Figure 3B**). There were no significant differences among any of the treatments in marketable yield for either ‘Florida Brilliance’ (*P* = 0.122) (**Figure 3C**) or ‘Florida Beauty’ (*P* = 0.206) (**Figure 3D**).

**Figure 4:**
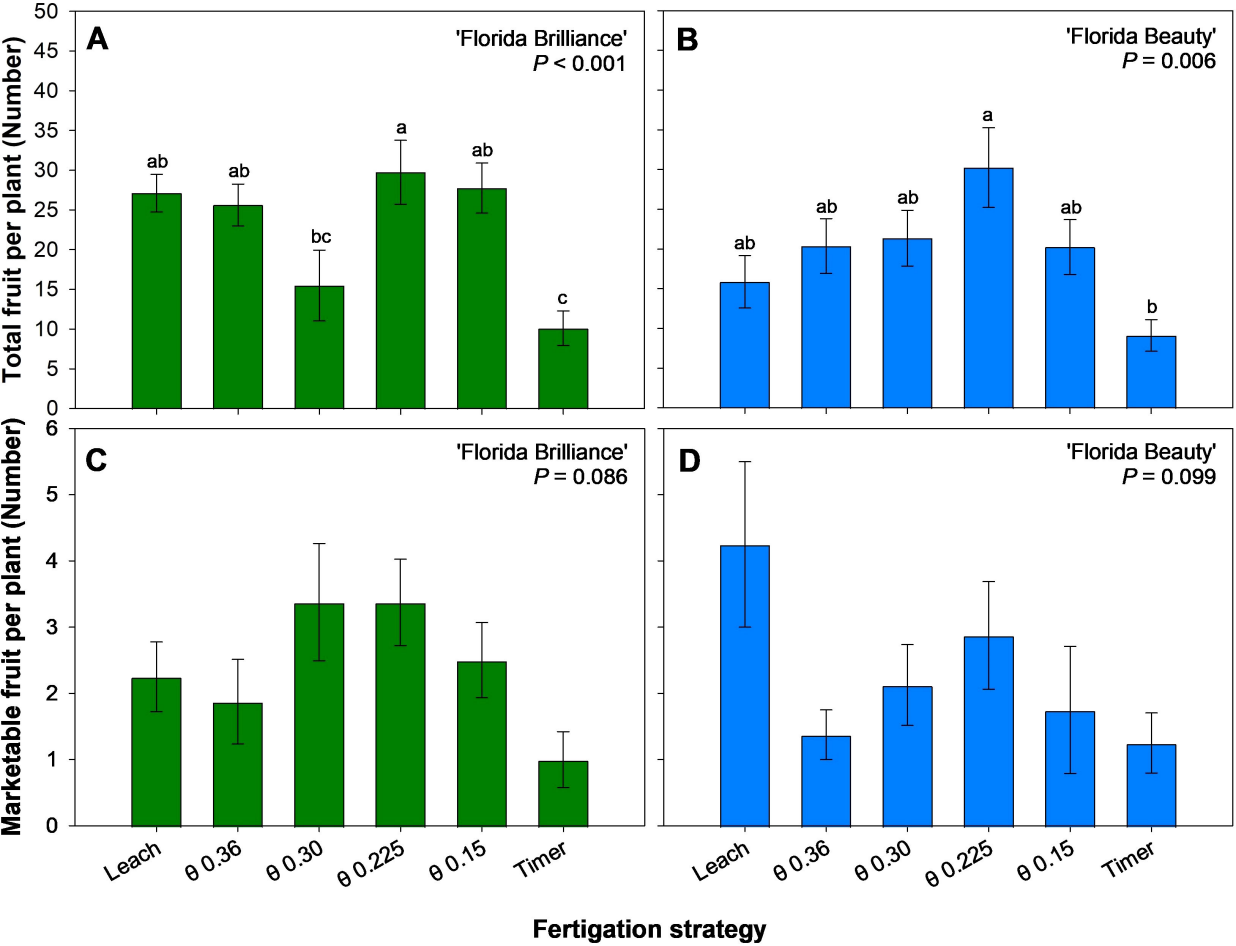
Total number of fruit per plant for ‘Florida Brilliance’ **(A)** and ‘Florida Beauty’ **(B)**, and number of marketable fruit per plant for ‘Florida Brilliance’ **(C)** and ‘Florida Beauty’ **(D)**. Each bar represents the average ± standard error of four replications with two measurement plants. Bars with the same letter show no significant difference; bars with different letters are statistically different at a 5% significance level (*P* < 0.05).

‘Florida Brilliance’ had at least 92% more fruit harvested from the θ 0.225 m^3^·m^-3^ treatment than from the Timer and θ 0.30 m^3^·m^-3^ treatments (**Figure 4A**). The Leach, θ 0.36, 0.225, and 0.15 m^3^·m^-3^ treatments also resulted in at least 153% more fruit harvested than the Timer treatment for the same cultivar (*P* < 0.001). The θ 0.225 m^3^·m^-3^ treatment resulted in 232% more fruit harvested than the Timer treatment in ‘Florida Beauty’, and this was the only significant difference among the treatments for this cultivar (*P* = 0.006) (**Figure 4B**). Similarly, there were no significant differences in the number of marketable fruit harvested among any treatments for ‘Florida Brilliance’ (*P* = 0.086) (**Figure 4C**) or ‘Florida Beauty’ (*P* = 0.099) (**Figure 4D**).

### 3.4 Fruit TSS, dry biomass, and water content

**Figure 5:**
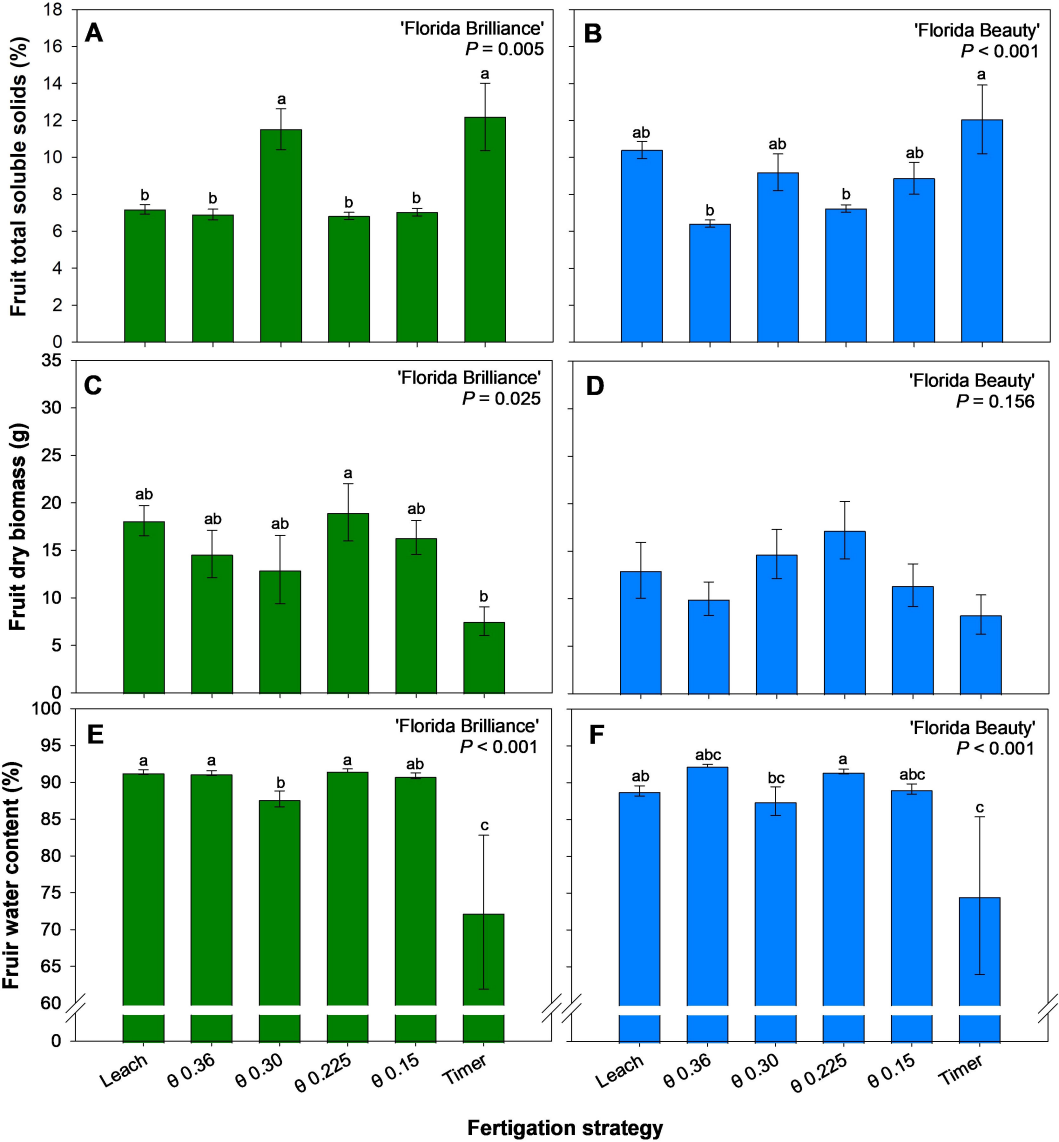
Fruit total soluble solids for ‘Florida Brilliance’ **(A)** and ‘Florida Beauty’ **(B)**, fruit dry biomass per plant for ‘Florida Brilliance’ **(C)** and ‘Florida Beauty’ **(D)**, and fruit water content for ‘Florida Brilliance’ **(E)** and ‘Florida Beauty’ **(F)**. Each bar represents the average ± standard error of four replications with two measurement plants. Bars with the same letter show no significant difference; bars with different letters are statistically different at a 5% significance level (*P* < 0.05).

The Timer and the θ 0.30 m^3^·m^-3^ treatments produced fruit with 44% higher TSS than the other treatments for ‘Florida Brilliance’ (*P* < 0.001) (**Figure 5A**). The Timer treatment also produced fruit with 40% higher TSS than the θ 0.36 and 0.225 m^3^·m^-3^ treatments in ‘Florida Beauty’ (*P* < 0.001) (**Figure 5B**). The θ 0.225 m^3^·m^-3^ treatment for ‘Florida Brilliance’ resulted in 152% higher fruit dry biomass than the Timer treatment (*P* = 0.025) (**Figure 5C**). There were no significant differences among the treatments for ‘Florida Beauty’ fruit dry biomass (*P* = 0.156) (**Figure 5D**). Similarly to the ‘Florida Brilliance’ results for fruit dry biomass, the Leach treatment, along with the θ 0.36 and 0.225 m^3^·m^-3^ treatments, resulted in at least a 26% increase in fruit water content compared to the Timer treatment for the same cultivar (*P* < 0.001) (**Figure 5E**). For ‘Florida Beauty’, the θ 0.225 m^3^·m^-3^ treatment showed at least a 23% increase in fruit water content compared to the Timer treatment (*P* < 0.001) (**Figure 5F**). Furthermore, the θ 0.225 m^3^·m^-3^ treatment showed at least a 3.6% increase in fruit water content compared to the θ 0.30 m^3^·m^-3^ treatment (*P* < 0.001).

### 3.5 Plant height, fresh shoot biomass, and dry shoot biomass

**Figure 6:**
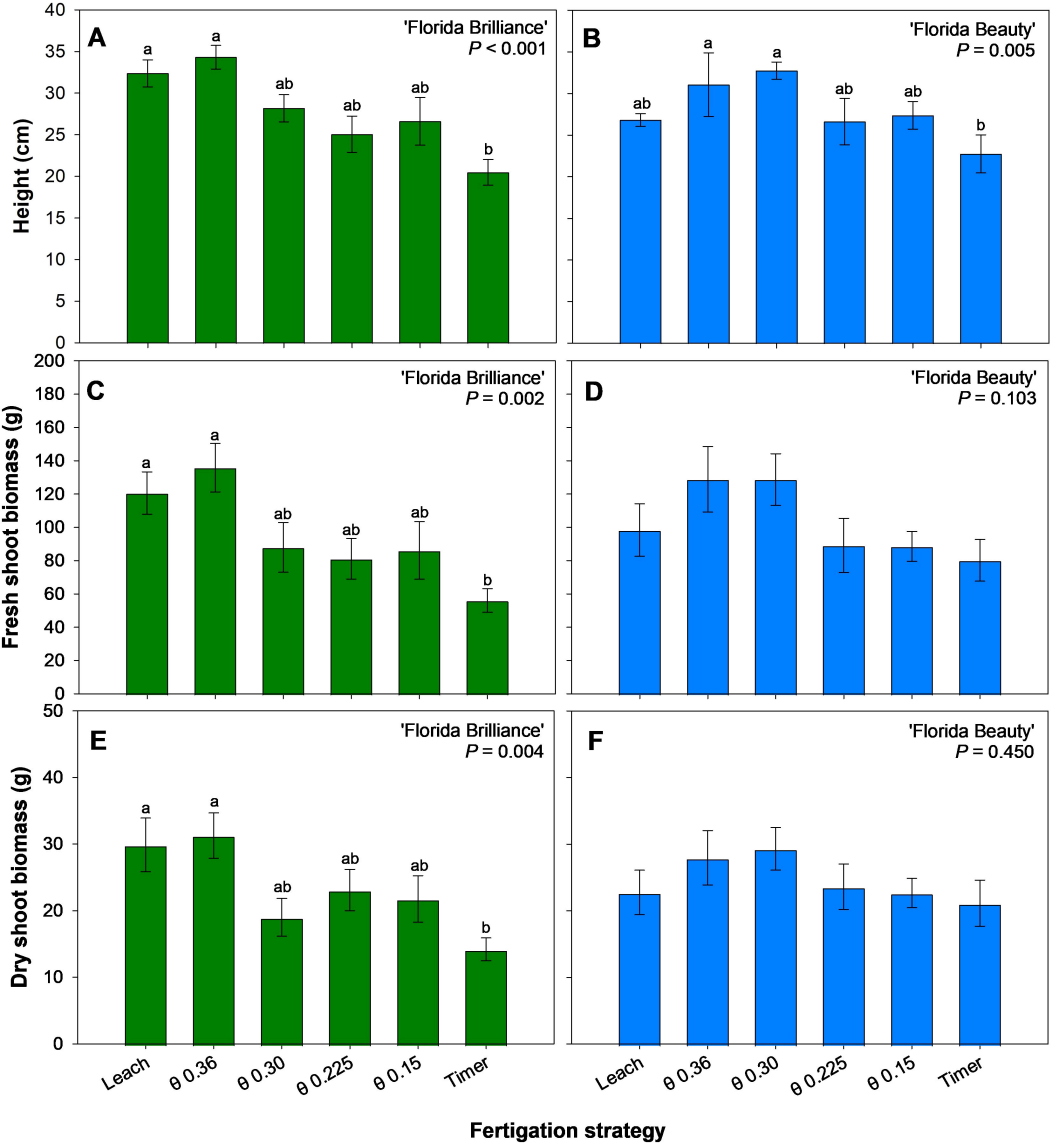
Plant height for ‘Florida Brilliance’ **(A)** and ‘Florida Beauty’ **(B)**, plant fresh shoot biomass for ‘Florida Brilliance’ **(C)** and ‘Florida Beauty’ **(D)**, and plant dry shoot biomass for ‘Florida Brilliance’ **(E)** and ‘Florida Beauty’ **(F)**. Each bar represents the average ± standard error of four replications with two measurement plants. Bars with the same letter show no significant difference; bars with different letters are statistically different at a 5% significance level (*P* < 0.05).

‘Florida Brilliance’ plants grew at least 58% taller under the θ 0.36 m^3^·m^-3^ and Leach treatments compared to plants under the Timer treatment (*P* < 0.001) (**Figure 6A**). ‘Florida Beauty’ plants grew at least 37% taller with the θ 0.36 and 0.30 m^3^·m^-3^ treatments compared to the Timer treatment (*P* = 0.005) (**Figure 6B**). Fresh shoot biomass for ‘Florida Brilliance’ was at least 115% higher in the Leach and θ 0.36 m^3^·m^-3^ treatment than in the Timer treatment (*P* = 0.002) (**Figure 6C**). ‘Florida Beauty’ fresh shoot biomass was not significantly affected by the fertigation treatments (*P* = 0.103) (**Figure 6D**). The results for dry shoot biomass were extremely similar to the fresh shoot biomass results for both cultivars. ‘Florida Brilliance’ had at least 110% higher dry shoot biomass in the Leach and θ 0.36 m^3^·m^-3^ treatments compared to the Timer treatment (*P* = 0.004) (**Figure 6E**). The treatments did not significantly affect the ‘Florida Beauty’ dry shoot biomass (*P* = 0.450) (**Figure 6F**).

### 3.6 Harvest index and leaf area

**Figure 7:**
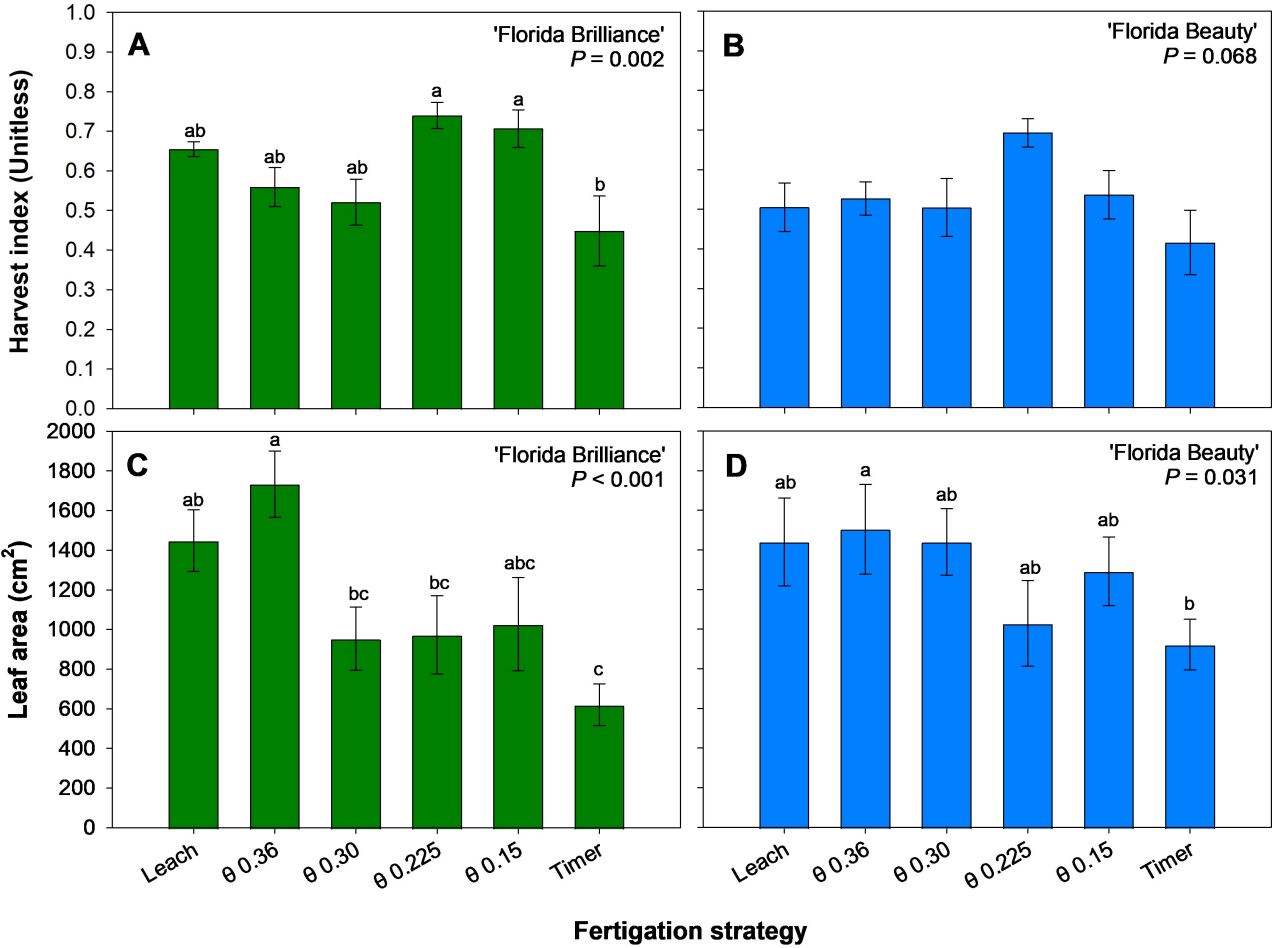
Harvest index for ‘Florida Brilliance’ **(A)** and ‘Florida Beauty’ **(B)**, and leaf area per plant for ‘Florida Brilliance’ **(C)** and ‘Florida Beauty’ **(D)**. Each bar represents the average ± standard error of four replications with two measurement plants. Bars with the same letter show no significant difference; bars with different letters are statistically different at a 5% significance level (*P* < 0.05).

‘Florida Brilliance’ harvest indices from the θ 0.225 and 0.15 m^3^·m^-3^ treatments showed at least a 57% increase from the Timer treatment harvest index (*P* = 0.002) (**Figure 7A**). The treatments did not significantly affect the ‘Florida Beauty’ harvest index (*P* = 0.068) (**Figure 7B**). The θ 0.36 m^3^·m^-3^ treatment for ‘Florida Brilliance’ resulted in at least 78% higher leaf area than the Timer treatment as well as the θ 0.30 and 0.225 m^3^·m^-3^ treatments (**Figure 7C**). The Leach treatment also resulted in a 133% higher leaf area than the Timer treatment for this cultivar (*P* < 0.001). The θ 0.36 m^3^·m^-3^ treatment resulted in 156% higher leaf area than the Timer treatment for ‘Florida Beauty’ (*P* = 0.184) (**Figure 7D**).

### 3.7 Leaf tissue macronutrient concentration

The fertigation management strategy treatments did not affect the ‘Florida Brilliance’ leaf N (*P* = 0.145) (**Table 1**). ‘Florida Brilliance’ leaf P was at least 47% higher in the θ 0.30 m^3^·m^-3^ and Timer treatments than the four other treatments (*P* < 0.001). Leaf K for ‘Florida Brilliance’ was 22% higher for the θ 0.30 m^3^·m^-3^ treatment than the Leach treatment (*P* = 0.020). The treatments did not affect the ‘Florida Brilliance’ leaf Mg (*P* = 0.060). ‘Florida Brilliance’ leaf Ca was 42% higher for the Timer treatment than for the θ 0.36 m^3^·m^-3^ treatment (*P* = 0.019). Finally, the treatments did not affect leaf S for ‘Florida Brilliance’ (*P* = 0.134). ‘Florida Beauty’ leaf N was 15% higher in the θ 0.30 m^3^·m^-3^ treatment than in the θ 0.15 m^3^·m^-3^ treatment (*P* = 0.016) (**Table 1**). Leaf P for ‘Florida Beauty was significantly affected by the treatments (*P* = 0.019), however, the *post-hoc* test could not distinguish between treatments. The treatments did not affect ‘Florida Beauty’ leaf K (*P* = 0.743). Leaf Mg for ‘Florida Beauty’ was 37% higher for the Leach treatment than for the θ 0.36 m^3^·m^-3^ treatment (*P* = 0.014). Leaf Ca for ‘Florida Beauty’ was 52% higher in the Timer treatment than in the θ 0.36 m^3^·m^-3^ treatment (*P* = 0.024). Finally, the treatments did not affect ‘Florida Beauty’ leaf S.

**Table 1:** ‘Leaf macronutrient concentrations of ‘Florida Brilliance’ and ‘Florida Beauty’ strawberries (*Fragaria × ananassa*) in response to the six fertigation management strategies.

Cultivar	Fertigation Management Strategy	N (%)	P (%)	K (%)	Mg (%)	Ca (%)	S (%)
‘Florida Brilliance’	Leach	2.38 ± 0.066	0.43 ± 0.025 b	2.31 ± 0.060 b	0.35 ± 0.020 ab	1.44 ± 0.057 ab	0.16 ± 0.005
	θ 0.36	2.48 ± 0.047	0.45 ± 0.018 b	2.38 ± 0.055 ab	0.30 ± 0.003 b	1.32 ± 0.066 b	0.16 ± 0.005
	θ 0.30	2.63 ± 0.135	0.66 ± 0.043 a	2.82 ± 0.111 a	0.37 ± 0.008 ab	1.70 ± 0.120 ab	0.18 ± 0.003
	θ 0.225	2.25 ± 0.077	0.42 ± 0.003 b	2.42 ± 0.077 ab	0.39 ± 0.010 a	1.72 ± 0.087 ab	0.17 ± 0.007
	θ 0.15	2.41 ± 0.036	0.43 ± 0.012 b	2.41 ± 0.152 ab	0.37 ± 0.008 ab	1.60 ± 0.081 ab	0.18 ± 0.010
	Timer	2.44 ± 0.139	0.67 ± 0.030 a	2.63 ± 0.114 ab	0.36 ± 0.023 ab	1.87 ± 0.176 a	0.17 ± 0.003
*P*		0.145	<0.001	0.020	0.060	0.019	0.134
Test		A	A	A	KW	A	KW
‘Florida Beauty’	Leach	2.55 ± 0.084 ab	0.66 ± 0.019 a	2.59 ± 0.088	0.41 ± 0.006 a	1.60 ± 0.126 ab	0.18 ± 0.005
	θ 0.36	2.52 ± 0.057 ab	0.45 0.019 a	2.43 ± 0.043	0.30 ± 0.013 b	1.32 ± 0.050 b	0.16 ± 0.003
	θ 0.30	2.60 ± 0.047 a	0.58 ± 0.059 a	2.55 ± 0.060	0.35 ± 0.017 ab	1.50 ± 0.099 ab	0.18 ± 0.008
	θ 0.225	2.37 ± 0.048 ab	0.45 ± 0.005 a	2.61 ± 0.073	0.37 ± 0.012 ab	1.60 ± 0.044 ab	0.18 ± 0.006
	θ 0.15	2.27 ± 0.082 b	0.55 ± 0.090 a	2.60 ± 0.239	0.39 ± 0.019 ab	1.66 ± 0.084 ab	0.17 ± 0.005
	Timer	2.31 ± 0.087 ab	0.73 ± 0.051 a	2.52 ± 0.055	0.37 ± 0.017 ab	2.00 ± 0.223 a	0.16 ± 0.000
*P*		0.016	0.019	0.743	0.014	0.024	0.055
Test		A	KW	KW	KW	A	KW

Leaf nitrogen (N) was determined by the high-temperature phosphorous (P), potassium (K), magnesium (Mg), calcium (Ca), and sulfur (S) were determined by inductively coupled plasma atomic emission spectrophotometer (ICP-AES) after wet acid digestion using nitric acid and hydrogen peroxide. Statistical analysis for leaf N, P, K, and Ca was conducted using one-way ANOVA and Tukey’s HSD (A), while the Kruskal-Wallis test with Dunn’s post-hoc (KW) was used for leaf Mg and S due to invalid ANOVA assumptions. Each value represents the average ± standard error of four replications with two measurement plants. Means with the same letter show no significant difference; means with different letters are statistically different at a significance level of 5% (*P* < 0.05).

### 3.8 Leaf tissue micronutrient concentration

‘Florida Brilliance’ leaf Zn was at least 38% higher in both the Timer and the θ 0.30 m^3^·m^-3^ treatments than in the other four treatments (*P* < 0.001) (**Table 1**). For ‘Florida Brilliance’ leaf Mn, the θ 0.225 m^3^·m^-3^ treatment was at least 135% higher than both the θ 0.36 m^3^·m^-3^ and Leach treatments. Furthermore, the θ 0.30 m^3^·m^-3^, θ 0.15 m^3^·m^-3^, and Timer treatments resulted in at least 179% higher leaf Mn than the θ 0.36 m^3^·m^-3^ treatment (*P* < 0.001). ‘Florida Brilliance’ leaf Fe was at least 121% higher in both the Leach and θ 0.225 m^3^·m^-3^ treatments than in the four other treatments (*P* < 0.001). Leaf B (*P* = 0.224) and leaf Cu (*P* = 0.354) were not affected by the treatments for this cultivar. ‘Florida Beauty’ leaf B was 44% higher in the Timer treatment than in the Leach treatment (*P* = 0.022) (**Table 1**). Leaf Mn for ‘Florida Beauty’ was 262% higher in the θ 0.225 m^3^·m^-3^ treatment than in the θ 0.36 m^3^·m^-3^ treatment (*P* = 0.006). ‘Florida Beauty’ leaf Fe was at least 447% higher in both the Leach and θ 0.225 m^3^·m^-3^ treatments than in the θ 0.30 m^3^·m^-3^ treatment (*P* = 0.002). Leaf Zn (*P* = 0.237) and leaf Cu (*P* = 0.311) were not affected by the treatments for this cultivar.

### 3.9 Resource use efficiency

**Figure 8:**
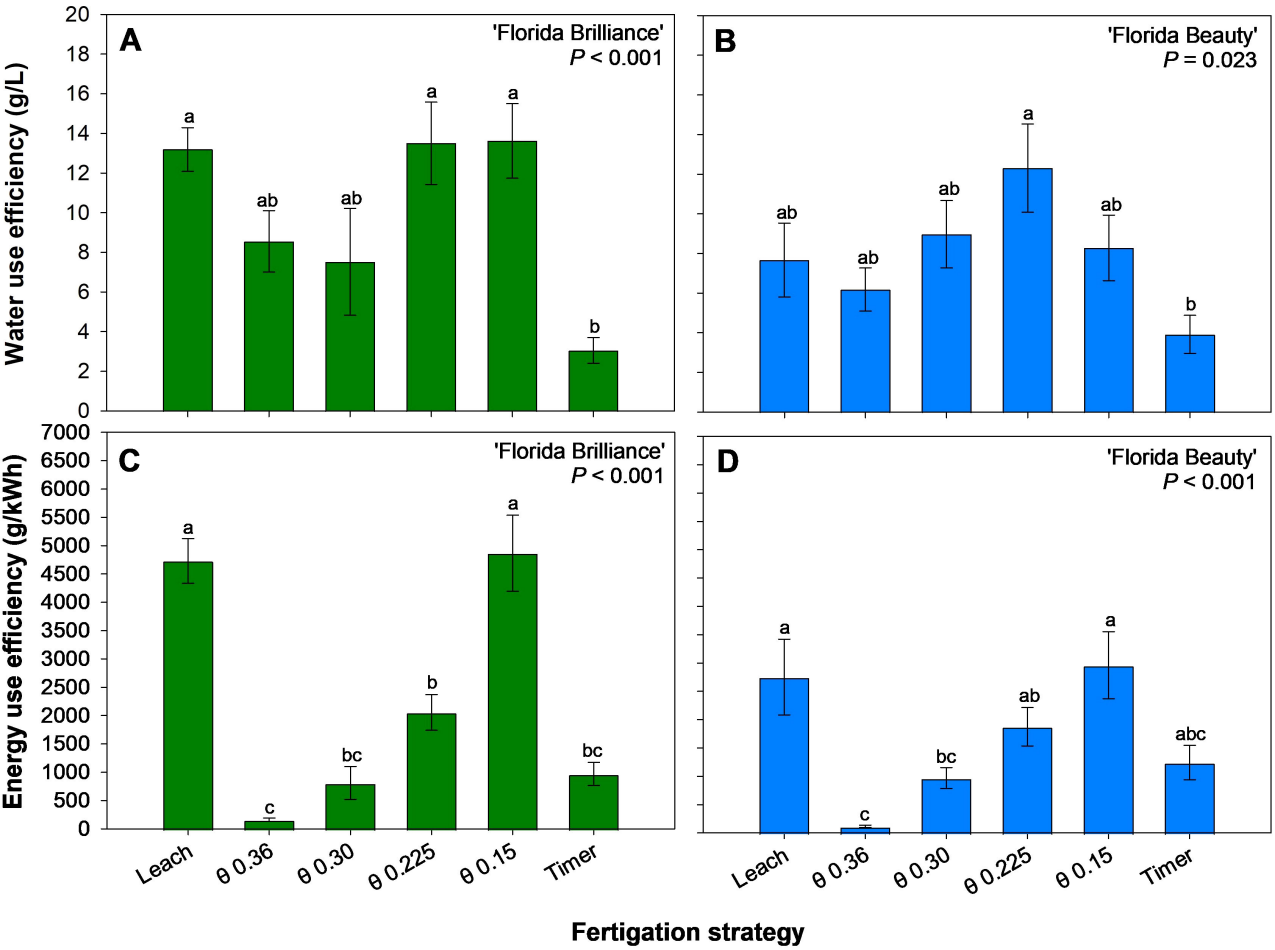
Water use efficiency for ‘Florida Brilliance’ **(A)** and ‘Florida Beauty’ **(B)**, and energy use efficiency for ‘Florida Brilliance’ **(C)** and ‘Florida Beauty’ **(D)**. Each bar represents the average ± standard error of four replications with two measurement plants. Bars with the same letter show no significant difference; bars with different letters are statistically different at a 5% significance level (*P* < 0.05).

WUE was at least 331% higher for ‘Florida Brilliance’ in the Leach, θ 0.225 and 0.15 m^3^·m^-3^ treatments than in the Timer treatment (*P* < 0.001) (**Figure 8A**). For ‘Florida Beauty’, the θ 0.225 m^3^·m^-3^ treatment resulted in 214% higher WUE than the Timer treatment (*P* = 0.023) (**Figure 8B**). ‘Florida Brilliance’ EUE was at least 389% higher in the Leach and θ 0.15 m^3^·m^-3^ treatments than in the other treatments (*P* < 0.001) (**Figure 8C**). ‘Florida Beauty’ EUE was at least 1,496% higher in the Leach and θ θ 0.15 m^3^·m^-3^ treatments than in the θ 0.36 m^3^·m^-3^ treatment (*P* < 0.001) (**Figure 8D**).

## 4 Discussion

### 4.1 Fruit yield and plant biomass

The Timer treatment resulted in reduced yields (**Figure 3**), reduced number of fruit per plant (**Figure 4**) as well as reduced vegetative biomass, including height, fresh shoot biomass, dry shoot biomass (**Figure 6**), and leaf area (**Figures 7C, D**) for both cultivars. This trend, especially regarding vegetative biomass, was more pronounced in ‘Florida Brilliance’ than in ‘Florida Beauty’. The Timer treatment also resulted in the highest fruit TSS in both cultivars (**Figures 5A, B**). This higher TSS is likely a result of the lower fruit water content in the Timer treatment (**Figures 5E, F**); as the water content decreases, solutes in the fruit become more concentrated, leading to a higher TSS. The Timer treatment being outperformed by sensor-based fertigation is not surprising, as previous studies have found similar results. One study reported a decrease in total and marketable yield for strawberries grown using timer-based fertigation compared to an FDR sensor-based fertigation (Bonelli et al., 2024). Another study observed a decrease in yield and plant fresh and dry biomass for strawberries grown using timer-based fertigation compared to one that used both solar irradiance sensors and soil moisture sensors to control fertigation automatically (Choi et al., 2021). Both of these previous experiments were conducted in greenhouses using soilless hydroponic substrate systems, and our study saw similar reductions from the Timer treatment compared to the sensor-based treatments.

In both cultivars, the θ 0.225 m^3^·m^-3^ treatment resulted in the highest total yield (**Figures 3A, B**), number of fruit (**Figures 4A, B**), and dry fruit biomass (**Figures 5C, D**). This treatment also resulted in the highest Mn and Fe foliar concentrations for both cultivars (**Table 2**). Mn is essential in the activation of several enzymes during the citric acid cycle, and it also aids in chlorophyll synthesis, nitrate assimilation, and the evolution of oxygen during photosynthesis. Fe is vital to the electron transport process during photosynthesis due to its inclusion in photosystem II, cytochrome *b6f*, photosystem I, and ferredoxin (Briat et al., 2015; Malone, 2014; Taiz and Zeiger, 2010). An increased foliar concentration of both essential micronutrients could have led to an increased availability of photosynthates, enhancing fruit growth and development. The increase in the concentrations of these two cations could be attributed to an improved cation exchange capacity due to the fertigation management strategy. Another possible explanation for the rise in yield, Mn, and Fe in the θ 0.225 m^3^·m^-3^ treatment is that this moisture level in the substrate could have been optimal for efficient and effective osmoregulation on a cellular level.

**Table 2:** Leaf micronutrient concentrations of ‘Florida Brilliance’ and ‘Florida Beauty’ strawberries (*Fragaria × ananassa*) in response to the six fertigation management strategies.

Cultivar	Fertigation Management Strategy	B (mg/kg)	Zn (mg/kg)	Mn (mg/kg)	Fe (mg/kg)	Cu (mg/kg)
‘Florida Brilliance’	Leach	151.50 ± 3.663	18.00 ± 0.816 b	187.75 ± 22.287 bc	514.00 ± 58.242 a	4.25 ± 0.250
	θ 0.36	139.00 ± 9.583	20.25 ± 0.854 b	102.50 ± 8.451 c	233.00 ± 12.994 b	3.75 ± 0.250
	θ 0.30	145.00 ± 9.600	30.75 ± 1.436 a	357.00 ± 65.029 ab	108.75 ± 15.024 b	4.25 ± 0.250
	θ 0.225	188.50 ± 11.064	22.25 ± 1.109 b	441.75 ± 24.095 a	559.50 ± 40.642 a	4.25 ± 0.629
	θ 0.15	170.25 ± 22.728	18.00 ± 1.472 b	285.50 ± 43.133 ab	230.50 ± 74.515 b	3.50 ± 0.289
	Timer	167.00 ± 21.973	32.00 ± 0.408 a	304.75 ± 50.954 ab	204.25 ± 67.288 b	4.25 ± 0.250
*P*		0.224	<0.001	<0.001	<0.001	0.354
Test		A	A	A	A	KW
‘Florida Beauty’	Leach	129.00 ± 11.881 b	23.00 ± 1.683	203.25 ± 9.801 ab	517.00 ± 86.374 a	4.00 ± 0.408
	θ 0.36	134.75 ± 10.641 ab	22.75 ± 1.250	115.75 ± 21.800 b	286.50 ± 48.550 ab	6.25 ± 1.931
	θ 0.30	158.00 ± 11.402 ab	22.25 ± 1.652	238.00 ± 63.005 ab	94.50 ± 9.242 b	4.25 ± 0.250
	θ 0.225	180.50 ± 12.301 ab	23.75 ± 1.887	419.50 ± 25.366 a	546.50 ± 59.389 a	3.75 ± 0.250
	θ 0.15	148.75 ± 16.540 ab	18.50 ± 2.327	228.50 ± 14.773 ab	247.50 ± 40.292 ab	3.75 ± 0.250
	Timer	185.50 ± 11.139 a	27.00 ± 1.780	284.50 ± 33.952 ab	163.25 ± 18.531 ab	3.75 ± 0.250
*P*		0.022	0.237	0.006	0.002	0.311
Test		A	KW	KW	KW	KW

Leaf boron (B), iron (Fe), copper (Cu), manganese (Mn), and zinc (Zn) concentrations were determined by inductively coupled plasma atomic emission spectrophotometer (ICP-AES) after wet acid digestion using nitric acid and hydrogen peroxide. Statistical analysis for leaf B, Zn, Mn, and Fe were conducted using one-way ANOVA and Tukey’s HSD (A), while the Kruskal-Wallis test with Dunn’s post-hoc (KW) was used for leaf Cu due to invalid ANOVA assumptions. Each value represents the average ± standard error of four replications with two measurement plants. Means with the same letter show no significant difference; means with different letters are statistically different at a significance level of 5% (*P* < 0.05).

Marketable yield was not affected by the treatments for either cultivar (**Figures 3C, D**). This is likely a result of poor pollination causing low marketable yields for all treatments. Thorough pollination of strawberry flowers is essential for fruit to develop a symmetric, marketable shape. Strawberry flowers can self-pollinate, but biotic pollinators have been shown to improve pollination and produce larger, more evenly shaped fruit (Gudowska et al., 2024). We could not add biotic pollinators inside the greenhouse for this study and thus relied on active (deliberate with hands and blowers) and passive (from ambient air circulation) mechanical pollination, which might have reduced marketable yields.

The wettest θ treatment (0.36 m^3^·m^-3^) resulted in either the highest or second highest plant height, fresh shoot biomass, dry shoot biomass (**Figure 6**), and leaf area (**Figures 7C, D**) for both cultivars. This is an expected result because several other studies have reported a positive relationship between θ and plant biomass accumulation in ornamental plants. Gaura (*Gaura lindheimeri*) has previously exhibited positive correlations between θ and both shoot dry weight and leaf area when grown at several different θ thresholds (Burnett and van Iersel, 2008). Hibiscus (*Hibiscus acetosella*) has also demonstrated positive correlations between θ and both shoot height and shoot dry weight under several different θ thresholds (Ferrarezi et al., 2015). We saw similar strawberry vegetative biomass accumulation increased under elevated substrate θ thresholds.

The treatment θ 0.225 m^3^·m^-3^ resulted in the most efficient biomass allocation, with approximately 70% of fresh biomass produced going to fruit rather than vegetative tissue in both cultivars (**Figures 7A, B**). This is a byproduct of this treatment resulting in slightly reduced fresh shoot biomass, with ‘Florida Brilliance’ having the second lowest and ‘Florida Beauty’ having the third lowest of the six treatments (**Figures 6C, D**) and the highest yields as previously described (**Figures 3A, B**).

### 4.2 Resource use efficiencies

The treatment θ 0.225 m^3^·m^-3^ also resulted in the highest WUE for ‘Florida Brilliance’ (**Figure 8A**) and ‘Florida Beauty’ (**Figure 8B**). The differences in WUE among treatments were driven primarily by yield since the magnitude of the difference in average water use among treatments is not excessive. The θ 0.15 m^3^·m^-3^ treatment had an average water use of 15 L per plant, the Timer 17.08 L per plant, the Leach 17.21 L per plant, the θ 0.225 m^3^·m^-3^ 17.8 L per plant, the θ 0.30 m^3^·m^-3^ 18.79 L per plant, and the θ 0.36 m^3^·m^-3^ 22.4 L per plant. Despite the θ 0.225 m^3^·m^-3^ treatment having the third highest average water use, the high yields from the treatment resulted in high WUE. Overall, the WUE results match very closely with the yield results for both cultivars. ‘Florida Brilliance’ yield and WUE were the highest in the Leach treatment and the two drier θ treatments: 0.225 and 0.15 m^3^·m^-3^, all of which had close outcomes for both variables. ‘Florida Beauty’ yield and WUE were the highest in the θ 0.225 m^3^·m^-3^ treatment, the only treatment significantly different from the Timer treatment for either variable. A previous study also reported higher WUE in an FDR sensor-controlled fertigation compared to a timer-based fertigation for ‘Seolhyang’ strawberries grown in coco coir (Choi et al., 2016). This indicates that, generally, a θ level of 0.225 m^3^·m^-3^ could produce high yields in hydroponic strawberry production and utilize water efficiently to produce those yields.

EUE for both cultivars (**Figures 8C, D**) was driven almost exclusively by the differences in average energy consumption among treatments. The θ 0.36 m^3^·m^-3^ treatment had the highest average energy consumption per plant of all treatments by an order of magnitude with 1.181 kWh. The θ 0.30 m^3^·m^-3^ treatment had the second highest with 0.174 kWh, the θ 0.225 m^3^·m^-3^ treatment had the third highest at 0.117 kWh, the Timer treatment had the third lowest at 0.054 kWh, the Leach treatment had the second lowest at 0.048 kWh, and the θ 0.15 m^3^·m^-3^ treatment had the lowest average energy consumption per plant at 0.042 kWh. The EUE results closely followed this pattern in both cultivars, except for the Timer treatment, which had reduced EUE due to lower yield.

## 5 Conclusion

These findings underscore the significant advantages of sensor-based irrigation strategies in optimizing yield and resource use efficiency, particularly when employing drier thresholds (θ 0.225 and 0.15 m^3^·m^-3^) that align with leaching fraction approaches. By demonstrating superior performance over traditional timer-based methods, this study highlights the potential of precision irrigation to enhance sustainable agricultural practices, paving the way for future advancements in dynamic sensor-based systems. Future research should explore dual- instead of single-threshold sensor-based strategies that emulate the wetting and drying cycle in the leaching-fraction strategy more closely.

## Data Availability

The raw data supporting the conclusions of this article will be made available by the authors upon reasonable requests.
